# A commentary on ‘Efficacy and safety of vitamin C for atrial fibrillation after cardiac surgery: a meta-analysis with trial sequential analysis of randomized controlled trials’

**DOI:** 10.1097/JS9.0000000000001269

**Published:** 2024-03-04

**Authors:** Changzhong Wang, Zijin Sun, Wei Zhou

**Affiliations:** a Graduate School of Tianjin University of Traditional Chinese Medicine; b School of Chinese Materia Medica, Beijing University of Chinese Medicine, Beijing,People’s Republic of China; c The Second Affiliated Hospital of Tianjin University of Traditional Chinese Medicine, Tianjin, People’s Republic of China

*Dear Editor,*

It was with great interest that we read the article published in the International Journal of Surgery. Hu *et al*.^1^ performed a meta-analysis with trial sequential analysis of randomized controlled trials on patients undergoing heart surgery who received vitamin C. Research suggests that short-term vitamin C therapy is safe and may lower the risk of postoperative atrial fibrillation following heart surgery.

Postoperative atrial fibrillation (POAF) is the most common arrhythmia after cardiac surgery, with an incidence ranging from 20 to 50% for patients undergoing coronary artery bypass grafting (CABG) to around 60% for those undergoing combined CABG and valve surgery^2^. Vitamin C may be a viable and successful treatment for postoperative atrial fibrillation (POAF) following heart surgery because of its antioxidant qualities. Heerman *et al*.^3^ conducted a retrospective cohort analysis on patients undergoing heart surgery who received vitamin C and discovered that there was no effect on LOS or the incidence of clinically meaningful POAF. Ghorbaninezhad *et al*.^4^ reported that tranexamic acid combined with vitamin C improved drainage (blood loss), but there was no significant difference in the incidence of AF between the two groups. Although findings are still mixed, the well-known antioxidant vitamin C is a potential and useful treatment for avoiding POAF.

The study also addresses trial sequential analysis (TSA), which is used to assess the validity and conclusiveness of the evidence to prevent repeated testing of accumulating data and sparse data from increasing the probability of type I errors in the meta-analysis. TSA provided sufficient and convincing evidence by demonstrating that the cumulative Z-curve exceeded the trial sequential monitoring barrier for benefit. Still, there were a lot of limitations. First, the nutritional state of the populations under study may have an impact on the study’s findings. For instance, a US study revealed that 20–30% of persons did not consume enough ascorbic acid. Second, the omission of potential confounders, such as varying perioperative antiarrhythmic therapy, could not be assured. Furthermore, the nutritional state of the populations under study may potentially have an impact on the findings of this and other studies.

This study uses TSA to assess if the information currently available was sufficient and conclusive in order to evaluate the safety and efficacy of vitamin C in preventing postoperative AF in adult patients following heart surgery. This issue has enormous ramifications for clinical practice. To determine the optimal course of treatment, further in-depth prospective research, clinical trials, and analysis of indications and risk factors in different patient subgroups are needed. Subsequent trials ought to concentrate more intently on outcomes that impact long-term and patient-relevant outcomes, like organ dysfunction, quality of life, discharge location, and ability to return to work^5^. More information should be provided about these clinically significant results, which include proxy measures of the various organ systems’ dysfunction since they may be more sensitive in determining the effectiveness of antioxidants or other micronutrients.

## Ethical approval

Not applicable.

## Consent

Not applicable.

## Source of funding

This work was supported by Tianjin Municipal Science and Technology Committee (2018KJ033).

## Author contribution

C.W.: study concept or design, data collection, data analysis or interpretation, and writing the paper; Z.S.: data collection; W.Z.: study concept or design, writing, and revise the paper.

## Conflicts of interest disclosure

The authors have no conflicts of interest to declare.

## Research registration unique identifying number (UIN)

Not applicable.

## Guarantor

Wei Zhou.

## Data availability statement

This manuscript is a comment. Do not need data availability statement. But all the data during the current study are publicly available.

## Provenance and peer review

This manuscript is a comment without being invited.
